# Effectiveness of a Web-Based Intervention to Prevent Anxiety in the Children of Parents With Anxiety: Protocol for a Randomized Controlled Trial

**DOI:** 10.2196/40707

**Published:** 2022-11-10

**Authors:** Abigail Dunn, James Alvarez, Amy Arbon, Stephen Bremner, Chloe Elsby-Pearson, Richard Emsley, Christopher Jones, Peter Lawrence, Kathryn J Lester, Mirjana Majdandžić, Natalie Morson, Nicky Perry, Julia Simner, Abigail Thomson, Sam Cartwright-Hatton

**Affiliations:** 1 Department of Psychology University of Sussex Brighton United Kingdom; 2 University Hospitals Sussex NHS Foundation Trust The Brighton and Sussex Clinical Trials Unit Brighton United Kingdom; 3 Brighton and Sussex Medical School Brighton United Kingdom; 4 Institute of Psychiatry, Psychology & Neuroscience King's College London London United Kingdom; 5 Department of Psychology University of Southampton Southampton United Kingdom; 6 Department of Child Development and Education University of Amsterdam Amsterdam Netherlands

**Keywords:** anxiety, parenting, online, RCT, child, parent, randomized controlled trial, youth, pediatric, mental health, mental well-being, online intervention, digital intervention

## Abstract

**Background:**

Anxiety is the most common childhood mental health condition and is associated with impaired child outcomes, including increased risk of mental health difficulties in adulthood. Anxiety runs in families: when a parent has anxiety, their child has a 50% higher chance of developing it themselves. Environmental factors are predominant in the intergenerational transmission of anxiety and, of these, parenting processes play a major role. Interventions that target parents to support them to limit the impact of any anxiogenic parenting behaviors are associated with reduced anxiety in their children. A brief UK-based group intervention delivered to parents within the UK National Health Service led to a 16% reduction in children meeting the criteria for an anxiety disorder. However, this intervention is not widely accessible. To widen access, a 9-module web-based version of this intervention has been developed. This course comprises psychoeducation and home practice delivered through text, video, animations, and practice tasks.

**Objective:**

This study seeks to evaluate the feasibility of delivering this web-based intervention and assess its effectiveness in reducing child anxiety symptoms.

**Methods:**

This is the protocol for a randomized controlled trial (RCT) of a community sample of 1754 parents with self-identified high levels of anxiety with a child aged 2-11 years. Parents in the intervention arm will receive access to the web-based course, which they undertake at a self-determined rate. The control arm receives no intervention. Follow-up data collection is at months 6 and months 9-21. Intention-to-treat analysis will be conducted on outcomes including child anxiety, child mental health symptoms, and well-being; parental anxiety and well-being; and parenting behaviors.

**Results:**

Funding was received in April 2020, and recruitment started in February 2021 and is projected to end in October 2022. A total of 1350 participants have been recruited as of May 2022.

**Conclusions:**

The results of this RCT will provide evidence on the utility of a web-based course in preventing intergenerational transmission of anxiety and increase the understanding of familial anxiety.

**Trial Registration:**

ClinicalTrials.gov NCT04755933; https://clinicaltrials.gov/ct2/show/NCT04755933

**International Registered Report Identifier (IRRID):**

DERR1-10.2196/40707

## Introduction

According to a systematic review of the prevalence of mental health conditions in childhood, anxiety disorders are more common than any other mental health disorder, including depression and behavior disorders [1]. However, this vulnerability is not randomly distributed; it is clear that anxiety disorders run in families and that children of parents with anxiety are at an increased risk of developing anxiety disorders themselves. A recent meta-analysis reported that the children of parents with anxiety were approximately twice as likely to have anxiety problems than those of parents without anxiety [2].

Moreover, it is clear that this intergenerational transmission of anxiety is not solely (or even largely) attributable to genetic processes; environmental factors have also been shown to contribute substantially to the transmission of anxiety within families [3]. The focus of the research that has sought to understand these environmental processes has been largely on parenting, and it seems likely that a number of parental processes are at play. For example, it has been reported that parents who experience significant anxiety show attentional biases to child-threat stimuli [4] and are more likely to show biased processing of ambiguous information about their children (for review, see [5]). In addition to these anxiety-based cognitive biases, there is evidence that the behavior of parents with anxiety is also altered in some situations. For example, parents with anxiety have been shown to encourage more avoidance and less approach in situations when their child is fearful [6]. Similarly, parents who experience high anxiety levels are more likely to report employing anxiogenic child behavior management techniques such as harsher discipline styles and inconsistency in following through on commands and on consequences for unacceptable behavior [7,8].

Children who develop anxiety disorders, regardless of their cause, are likely to face a number of challenges: they experience lower quality of life than other children [9], and although some will become less anxious as they grow older, a large proportion will experience anxiety-related difficulties into adulthood [10]. Many will also experience educational difficulties [11] and difficulties with relationships [12]. Children with anxiety disorders are also at an increased risk of developing other ailments, particularly mood disorders [13] and substance use disorders [14]. Moreover, childhood anxiety disorders are costly to society: a Netherlands-based study found that the societal costs associated with anxiety disorders in children were 21 times higher than those for children without anxiety disorders [15].

Despite evidence that children of parents with anxiety are at an increased risk of anxiety disorders and the problems that anxiety disorders bring, little has been done to prevent this intergenerational transmission. Ginsburg et al [16] sought to address this gap, devising the US-based “Coping and Promoting Strength” program for parents who experience high levels of anxiety and their children. The intervention comprised a 60-minute session each week for 8 weeks, followed by 3 optional booster sessions each month, and employed a range of psychoeducational, cognitive restructuring and problem-solving approaches to cover both child- and parenting-related factors. Although the program produced encouraging results, it is unlikely to be viable in the financial and operational context of the National Health Service (NHS) in the United Kingdom (and other similar systems). Furthermore, it is onerous for families; if their children are currently doing well, it may seem particularly burdensome.

In an attempt to address these constraints, Cartwright-Hatton et al [17] developed a very brief, clinic-based group intervention aimed at parents who were seeking treatment for their own difficulties with anxiety. The aim of this 1-day intervention was to help parents develop a calm, consistent behavior management style, while learning skills to discourage children’s avoidance and encourage confident behaviors. During the sessions, parents were also supported to identify areas where anxiety might affect their parenting and to make plans to minimize this. A UK NHS–based randomized controlled trial of this intervention reported 16.5% fewer children meeting the criteria for an anxiety disorder 1 year later, compared to those whose parents did not receive the intervention [17].

It is clear that there are ways in which the intergenerational transmission of anxiety can be reduced. However, the two aforementioned currently available evidence-based interventions have the capacity to reach only a tiny fraction of the families that might benefit. For example, in the United Kingdom, primary care mental health services (where such interventions would likely be based) aim to reach only 25% of people with clinically diagnosed anxiety, of whom only 50% are expected to meet the criteria for recovery after treatment (NHS England, 2022 [18]). The children of the estimated 75% who do not access support for their anxiety are likely to be as vulnerable (if not more so) as those whose parents do receive some support. Moreover, even the 25% of adults who do access assistance for their anxiety may not have the resources to attend an additional in-person intervention aimed at the needs of their children. In the randomized controlled trial conducted by Cartwright-Hatton et al [17], and in their clinic offering the intervention since, approximately half of interested parents fail to attend as a result of childcare or work commitments or because a group-based intervention was unacceptable. Therefore, this paper describes the protocol for a randomized controlled trial of a self-guided, web-based version of Cartwright-Hatton’s [17] intervention. If effective, this digital intervention has the potential to increase access to a substantially larger group of parents with anxiety, including those who do not or cannot access mental health services, and those who cannot attend a workshop in person.

Fathers are particularly disadvantaged by these barriers to accessing mental health services and clinics. This may be attributable in part to the traditional family model of the mother as the primary caregiver and father as the main earner; that is, it is assumed that attending to children’s health and emotional needs is part of the mother’s role and not part of the father’s domain. This is supported by the fact that parenting interventions are overwhelmingly focused on mothers: interventions tend to concentrate on the recruitment of mothers and consider what would be most convenient to them. Panter-Brick et al’s [19] systematic review of fathers’ inclusion in parenting interventions found that the vast majority of studies included few or no fathers, with very little data disaggregated by parent gender. However, focusing mainly on mothers in psychological interventions is problematic. There is clear evidence that fathers have a key role to play in children’s emotional development, and greater involvement from fathers in parenting has been shown to have positive outcomes for children [20]. Moreover, in terms of the transmission of anxiety, it is possible that fathers who experience significant anxiety might have different and potentially more deleterious impacts on child outcomes than do mothers with anxiety [21,22]. It is hoped that in providing this intervention in a self-guided, digital format, more fathers will be reached.

We believe that there is value in increasing confidence and reducing anxiety in both a child who is only at risk of subthreshold anxiety and in reducing the severity of symptoms in a child with an ongoing anxiety disorder. The study, therefore, takes a “public health” approach; that is, the intervention is intended not only to reduce the risk of clinically diagnosable anxiety disorders but also to reduce the likelihood of symptoms across the anxiety spectrum. 

Similarly, we do not believe that the intervention might be of benefit only to children whose parents have clinically diagnosable anxiety disorders; hence, we will invite any parent who identifies as experiencing high levels of anxiety to participate in the study without requiring a formal diagnosis of anxiety. By sampling into the subclinical range of parental anxiety, we will maximize preventative opportunities. This may be of particular relevance given the heightened level of anxiety experienced by many parents during the course of the COVID-19 pandemic [23].

Research into preventing anxiety disorders is in its infancy, and this is particularly the case for efforts that are targeted among parents with anxiety. While there are reasonable models of the processes involved in the intergenerational transmission of anxiety, little is known about what changes in parenting behavior are necessary and sufficient for preventative interventions to be effective. To maximize efficiency for parents (and services), we need to understand which intervention components are most effective, and for whom, particularly in a web-based format and for the wider, more inclusive group that is likely to access this. This study aims to recruit a very large sample of parents, which will allow us to conduct an analysis of intervention components. In doing so, we will not only optimize the intervention but also gain a better theoretical understanding of the mechanisms underpinning intergenerational transmission of anxiety.

## Methods

### Study Aims and Objectives

This study aims to evaluate the effectiveness of a web-based intervention designed to prevent anxiety in the children of parents with anxiety and provide information for the optimization of this intervention. The study has 3 core objectives:

1. To investigate the effectiveness of a web-based, parent-focused intervention for the prevention of anxiety in children of parents with anxiety.
2. To determine which components of the intervention have the most or least impact on outcomes and test whether the effect of each component is moderated by participant characteristics (type or severity of parent or child anxiety symptoms, socioeconomic status, and child age).
3. To explore the impact of coparent anxiety and parenting behaviors on child outcomes—we hypothesize that child outcomes will be worse when the coparent is also anxious or engages in frequent anxiogenic parenting behaviors.

### Study Setting

The study will be completed entirely in a digital setting, with UK-based, self-referred participants.

### Sample Size

The sample size was calculated to provide adequate power for our first objective (to detect a difference between trial arms in child anxiety). Based on existing research carried out by the trial team, a small effect size is anticipated (Cohen *d*=0.2) [17]. With 90% power for 5% significance, this requires 526 participants in each of 2 arms of the trial. Allowing 40% attrition, which is likely to be substantial in a web-based study, we need to randomize 877 participants to each arm, or 1754 in total.

### Eligibility Criteria

The eligibility criteria are designed to resemble those that would be employed in any eventual rollout of a web-based intervention. As such, they are minimal. To be eligible, a participant must be a parent (any gender; adoptive, biological, step, foster, or grandparent) residing in the United Kingdom, aged over 16 years and with a child aged 2 to 11 years (inclusive). The participant must have at least 50 days’ contact with the index child per year and confirm that they see enough of the child to report on the child’s current anxiety level. The participant must self-report subjectively substantial levels of current or lifetime anxiety (it is not necessary to have a diagnosis) and be able to commit to completion of measures at (up to) 3 time points, even if allocated to the control arm.

Participants will not be excluded on the basis of current or previous psychiatric treatment (parent or child) or on any psychological, neuropsychological, or physical condition.

### Recruitment, Randomization, and Allocation

This study is intended to reach parents with anxiety, the majority of whom never receive treatment for anxiety (although those who have will not be excluded). As such, recruitment activities will focus outside of health services, and will involve the dissemination of study materials by mental health charities, schools, parent groups, parenting magazines, social media, and through the Genetic Links to Anxiety and Depression (GLAD) study [24] within the National Institute for Health and Care Research (NIHR) Mental Health Bioresource.

To reach potential participants directly, we will utilize social media platforms, including contracting a public relations agency to run a paid-for social media campaign. We will also commission advertisements in parent-orientated publications. To maximize the recruitment of fathers, we will partner with male mental health organizations and organizations supporting fathers including charities and social media influencers.

Participants will be randomized in large blocks using block randomization to 1 of 2 arms (intervention or control [no Intervention]) in a ratio of 1:1. This will be carried out using predefined lists generated by the Brighton and Sussex Clinical Trials Unit, with study IDs all prerandomized and concealed until the participant completes baseline questionnaires. Those assigned to the intervention group will be further randomized to one of 8 conditions to allow analysis of the intervention components (see *Interventions* below). If a parent has more than one child within the target age range (2-11 years), the web-based system will allocate one child at random on which the parent will report when responding to outcome measures.

Given the digital nature of the intervention and use of self-report measures, no assessor or clinician blinding is required.

### Interventions

Eligible participants will be randomized in equal proportions between the intervention arm, which comprises a web-based parenting course, and the control arm, which receives no provision over and above what the parent might be accessing outside of the study.

The parenting course is delivered fully digitally and follows the format of the evidence-based in-person version [17]. It has one “Starter” module and 8 “further” modules and is completed by parents (children do not participate). The Starter module is completed by all parents. Subsequently, parents complete 7 of 8 further modules (one is disabled at random), which are displayed to parents in random order. The modules cover the following subject areas: the Seven Confident Thoughts, avoidance, play, emotion coaching, positive behavior management, basic needs, hotspots and overprotection, and modeling and compensation. Each module takes between 20 and 30 minutes to compete and has accompanying home practice tasks, which the participant is encouraged to try out before progressing to the next module. An overview of the course module content and example intervention images can be found in Multimedia Appendix 1 and Multimedia Appendix 2.

Participants can share a “mirrored” version of the course with a supporter to facilitate shared learning and support. To promote adherence, participants will be automatically “nudged” with a limited number of email and SMS text messages should they disengage from the intervention for a period in excess of 73 hours if they have partially completed a module, and 1 week if they are between modules.

The intervention may be modified during the study, only on the grounds of external activities (eg, an amendment to mobile operating systems) or should disproportionate attrition be identified in one or more modules.

### Participant Timeline

The entire study will be completed digitally. Participants will flow through the study as follows:

1. Receive summary information.
2. Be screened for inclusion and exclusion criteria (see Eligibility Criteria above). Those meeting the criteria will proceed to step 3.
3. Receive detailed information about the study and give consent digitally.
4. Invited to provide details of a corespondent who will be asked to complete a measure of child anxiety. This person, who can be a coparent, friend, or family member who has good knowledge of the index child, will be emailed from within the study platform 48 hours after the participant has completed measures (allowing parents time to explain the study to their chosen corespondent). Participants can also choose not to refer or to make a referral later.
5. Participants (and corespondents, if applicable) complete baseline measures.
6. Participants will be informed of the arm of the trial they have been randomized to.
7. Undertake intervention or control group tasks.
8. Participants (and corespondents, if applicable) complete outcome measures at 6 months (same outcomes as the baseline assessment).
9. Participants (and corespondents, if applicable) recruited prior to the final 6 months of recruitment will complete outcome measures again, once, 4 months prior to the study end to allow exploration of longer-term outcomes. This follow-up is contingent on the participant submitting their 6-month follow-up questionnaire a minimum of 2 months prior to this point.

**Figure 1:**
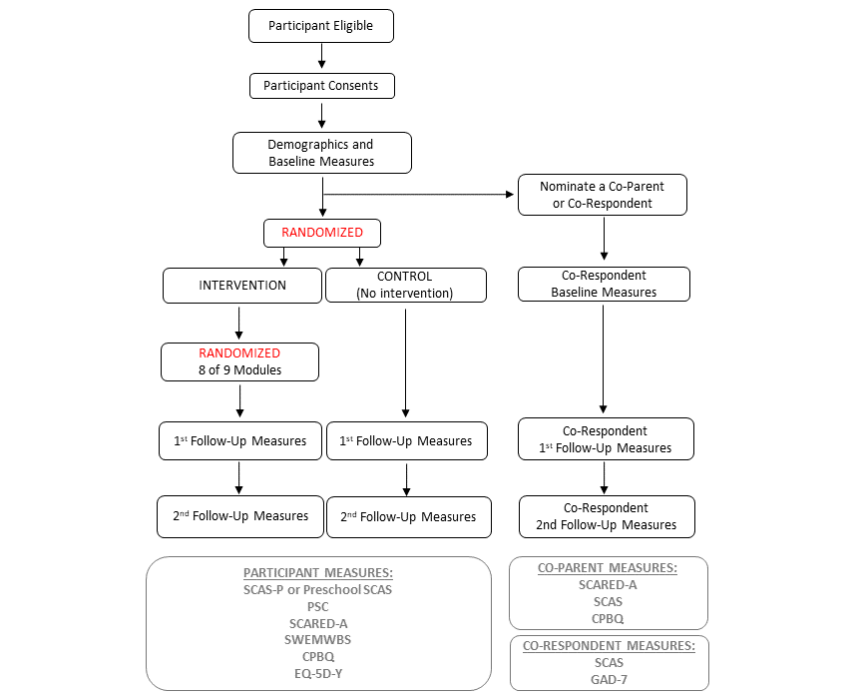
Participant flow through the project. CPBQ: Comprehensive Parenting Behavior Questionnaire; PSC: Pediatric Symptom Checklist; SCARED-A: adult version of the Screen for Child Anxiety Related Disorders; SCAS: Spence Children’s Anxiety Scale; SCAS-P: parent-report version of the Spence Children’s Anxiety Scale; SWEMWBS: Short Warwick Edinburgh Mental Well-being Scale.

Participants have the option of inviting a corespondent to also complete a measure of child anxiety, allowing us to access a different perspective on the child. Where the corespondent is a coparent, they will additionally be asked to complete a measure of their own anxiety and of parenting behavior, which will be used in support of objective 3: to explore the impact of coparent anxiety and parenting behaviors on child outcomes. Corespondents will be invited to complete follow-up measures at 6 months post consent and 4 months prior to the end of the trial. Participant flow through the study is shown in Figure 1.

### Outcomes

Unless otherwise indicated, “outcomes” are those reported by the index parent, in both arms, at baseline, at 6 months, and in the final follow-up.

To determine the effectiveness of the web-based parenting-focused intervention, the primary outcome will be child anxiety symptoms. The secondary outcomes will be parental anxiety and parental well-being. We hypothesize that children and parents in the intervention arm will show fewer anxiety symptoms (and greater parental well-being) at 6-month follow-up than those in the control arms.

Intervention feasibility and acceptability will be examined on the basis of the following parameters: study attrition rate, which is the proportion of participants that complete 6-month follow-up measures; study completion rate, which is the proportion of participants in the intervention arm who complete 3 modules; and participants’ self-reported satisfaction with the intervention modules.

### Data Collection, Management, and Analysis

#### Primary Outcome

Child anxiety symptoms will be assessed using the Spence Children’s Anxiety Scale (SCAS-P [parent-report version of the SCAS] and Preschool SCAS) [25,26]. These parallel instruments are acceptable to parents and have good validity or reliability.

#### Secondary Outcomes

##### Parent and Coparent Anxiety Symptoms

The adult version of the Screen for Child Anxiety Related Disorders (SCARED) [27] will be used to assess each of the Diagnostic and Statistical Manual of Mental Disorders (DSM) anxiety disorders, consisting of 71 items. It has good internal consistency and is positively correlated with results from the diagnostic anxiety disorders interview schedule of the Anxiety Disorders Interview Schedule for DSM-IV: Lifetime version.

##### Parent Well-being

The Short Warwick Edinburgh Mental Well-being Scale [28], a 7-item self-report measure (rated 1 to 5) of features of positive mental health (positive affect, interpersonal relationships, and positive functioning), will be used. It is positively correlated with the original 14-item Warwick-Edinburgh Mental Well-being Scale [29] and has high internal consistency and good validity.

##### Child Emotional and Behavioral Symptoms

The Pediatric Symptom Checklist, a 17-item general mental health screening tool for children, will be used. The parent reports how often their child demonstrates internalizing, externalizing, or attentional symptoms using 3 levels of frequency. The scale has good validity and sensitivity with comparable case detection to semistructured clinical interviews and is widely used as a screen in primary care [30].

##### Child Health

We will use the EQ-5D-Y Proxy Report [31], a 5-item plus visual analogue scale proxy report measure of health-related quality of life, developed for use with children. The parent reports how they would measure the child’s health (on that day) across 5 dimensions—mobility; looking after themselves; doing usual activities; having pain or discomfort; and feeling worried, sad, or unhappy—using 3 levels of severity, and further marks “health today” on a scale of 0 to 100. This is a version of the EQ-5D-3L, which is widely used in cost-effectiveness analyses and has good validity and internal consistency.

##### Demographics

Parents’ age, gender, socioeconomic status, ethnicity, coparenting status, child age, developmental disability, and previous parent or child treatment for anxiety will be assessed.

##### Adverse Events

A short instrument has been designed for the study; it is based on the number of children and parents whose anxiety deteriorates.

##### Mediators

We will measure parenting behavior by asking parents (in both arms) to complete measures of parenting behaviors that are addressed by each module. For most modules, items are taken from a 104-item shortened version of the Comprehensive Parenting Behavior Questionnaire (CPBQ) [32,33], a psychometrically strong, self-report instrument measuring parenting behaviors associated with the risk of child anxiety. For the few areas in our intervention not covered by the CPBQ (eg, sleep, exercise, and diet), items have been constructed for the purposes of this study.

##### Moderators

We will assess the following baseline parameters and changes in them, where appropriate: severity or type of parent and child anxiety (SCAS and adult version of the SCARED), socioeconomic status, previous parent or child treatment for anxiety, parent and child gender, child age, and child developmental disabilities.

Maximizing response rate and retention of participants is a priority, so participants and corespondents will be offered shopping vouchers for completion of measures. Participants will be provided with a £15 (US $17.39) voucher on completion of follow-up measures; corespondents will be provided a £10 (US $11.59) voucher on completion of all measures.

### Data Management

Data will be entered directly by participants via Moodle, a secure web-based learning platform and via an embedded survey hosted by Qualtrics. Upon randomization, the participants will each be given a unique identifier by which they will be referred for the duration of the study. This entire database is encrypted. In order to collect special category data (eg, mental health data and ethnicity), we have in place a Secure Sockets Layer setup, which will establish an encrypted link between a web server and a browser. All investigators will comply with the requirements of the Data Protection Act of 1998 [34]. A specific data management plan and a monitoring plan have been developed for the study (available from Brighton and Sussex Clinical Trials Unit).

### Data Analysis

#### Overview

We will report participant flow through the trial and results in line with the 2010 Consolidated Standards of Reporting Trials statement [35]. All analyses will be carried out following intention-to-treat principles, incorporating data from all participants in their allocated arm, including those who do not complete the intervention.

#### Primary and Secondary Outcome Analysis

##### Objective 1

Descriptive statistics will be presented by randomization arm. At baseline and 6 months, these will include counts and percentages for binary and categorical variables and means and SDs or medians with lower and upper quartiles for continuous variables and counts of missing values. Number of adverse events will be presented as the number of events and number of individuals with events by randomization arm and in accordance with the treatment received. We will also report the number of parents in each arm whose anxiety worsens.

For primary and secondary outcomes, we will analyze using multiple linear regression and include a fixed effect for intervention versus control groups, adjusting for baseline child anxiety severity. Other covariates considered—a priori, to be prognostic of outcome at 6 months (particularly parent gender)—may be included in the linear models and written into the analysis plan prior to sign off. Treatment effects (between-group differences) will be reported as the adjusted mean difference with 95% CIs. Cohen *d* effect sizes at 6 months will be calculated as the adjusted mean difference of outcomes divided by the sample SD of the outcome at baseline. Potential moderators will be assessed by including the randomized arm by moderator interactions as fixed effects.

##### Objective 2

To examine the contribution of each component of the intervention, we will compare those randomized to the intervention containing a given module (eg, module E), to those randomized to the intervention but not containing that component, and to those randomized to the control arm (ie, no intervention). Using the measure of parent behavior congruent with each module as a dependent variable, we will test whether there are differences between these groups each for modules A to H separately (parent behavior measures are shown in Multimedia Appendix 3). For all modules, we will perform a mediation analysis with the primary outcome as the dependent variable and the measure of parenting behavior congruent with that module as a mediator.

##### Objective 3

The analysis conducted to explore the impact of coparent anxiety and parenting behaviors on child outcomes will be restricted to participants where the coparent agrees to provide data. We will include baseline covariates (their anxiety type and severity and parenting variables) in a linear regression model with randomization as a fixed effect and child anxiety severity along with other variables to be agreed prior to database lock at 6 months as the outcome variable [36]. For scaling data with actively unanswered items, we will either prorate or perform item-level multiple imputation [37].We will perform multiple imputation if we can identify predictors of missingness upon fitting logistic regression models that include variables not present in the analysis models, which may be variables collected post randomization.

Where outcome data are missing (assumed to be missing at random), we will perform multiple imputation using chained equations to create (eg, 10) completed data sets, the analyses of which will be pooled using Rubin’s Rules.

A detailed statistical analysis plan will be agreed on prior to final analysis. Analyses will be conducted in Stata (version 17.0 or later; StataCorp) [38].

### Ethics Approval

The study has been approved by the University of Sussex Cross Schools Research Ethics Committee (ER/SC430/1) and is registered on ClinicalTrials.gov (NCT04755933) [39]. Peer reviews of the study issued as part of the grant application process can be accessed in Multimedia Appendix 4.

## Results

The study was funded in April 2020. Recruitment started in February 2021 and is projected to end in October 2022. A total of 1350 participants have been recruited as of May 2022.

## Discussion

This paper describes the protocol for a randomized controlled trial to test the effectiveness of a web-based intervention for parents with anxiety. We hypothesize that children and parents in the intervention arm will show significantly fewer anxiety symptoms (and greater parental well-being) at 6-month follow-up than those in the control arm. Given the extensive data set generated in the study, it is anticipated that the final results will offer valuable evidence on the utility of a web-based course in preventing intergenerational transmission of anxiety, as well as broader evidence regarding familial anxiety, anxiogenic parenting behaviors, and intergenerational transmission.

The findings from this study will be disseminated as follows. Outcomes will be delivered to academic audiences via fully open access journals and conferences (including user conferences). Anonymized data will be entered into a data repository at the end of the study (in liaison with steering and ethics committees). The results will be communicated to the participants and the wider public via a short video. A Plain English summary of the results will be disseminated to all stakeholders and participants and will be published on the study website.

## Appendix

Multimedia Appendix 1 Digital intervention content.

Multimedia Appendix 2 Intervention images.

Multimedia Appendix 3 Intervention measures.

Multimedia Appendix 4 Peer review report by The Kavli Trust Programme on Health Research (Bergen, Norway).

## Abbreviations

CPBQ: Comprehensive Parenting Behavior Questionnaire
DSM: Diagnostic and Statistical Manual of Mental Disorders
GLAD: Genetic Links to Anxiety and Depression
NHS: National Health Service
NIHR: National Institute for Health and Care Research
SCARED: Screen for Child Anxiety Related Disorders
SCAS: Spence Children’s Anxiety Scale

## Data Availability

At the end of the study, anonymized data will be entered into a Figshare data repository [40].
