# Supplementary material for: Effectiveness of a Web-Based Intervention to Prevent Anxiety in the Children of Parents With Anxiety: Protocol for a Randomized Controlled Trial
Source: JMIR Res Protoc. 2022 Nov 10;11(11):e40707. doi: 10.2196/40707 (PMC9693706; doi:10.2196/40707)
Supplement: Multimedia Appendix 2 [file resprot_v11i11e40707_app2.pdf]

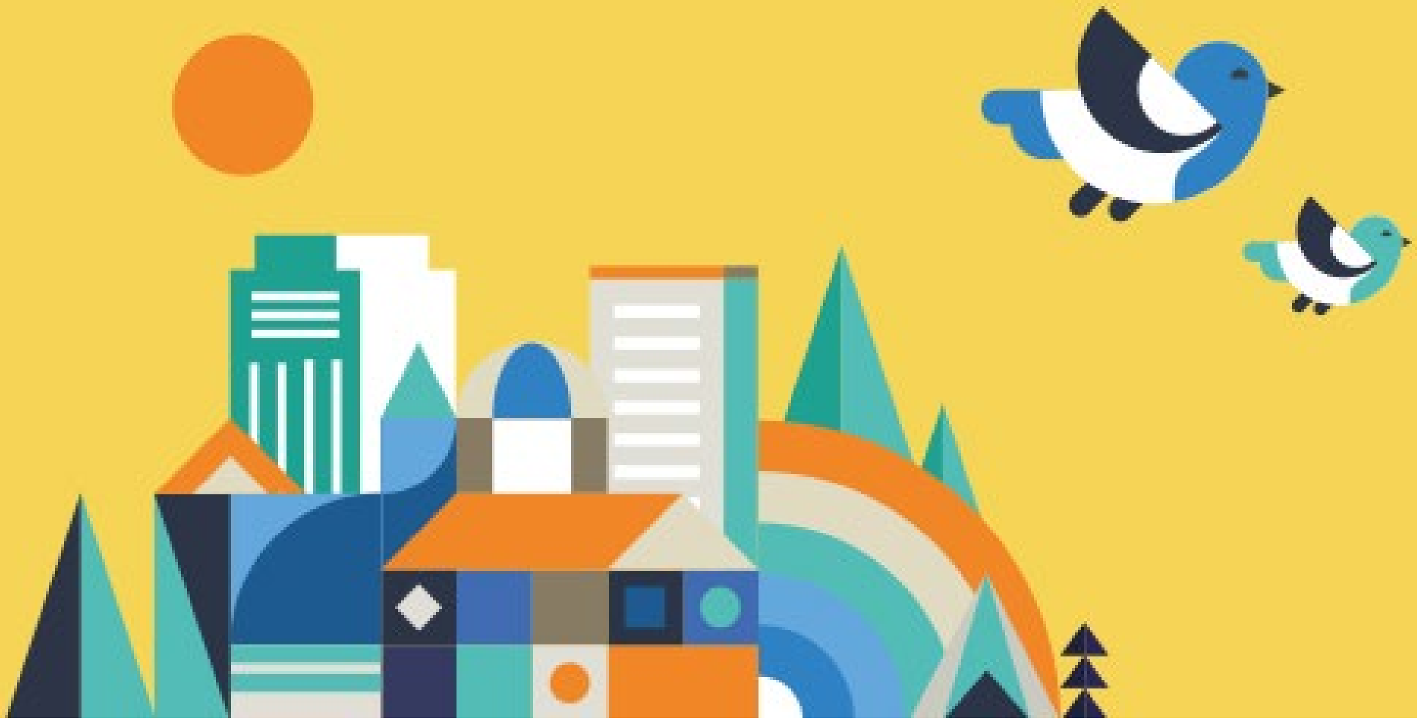

Parenting with Anxiety: intervention images

≡ PWA

Abby Dunn

- Raising Confident Children
- Badges
- My Summary Notes and Action Plans

Dashboard

Start your course!

Click here

Invite Friend

It can be useful to share the contents of this course with your partner, friend or relative so that they can support you as you work through the modules. You can invite one person to access the course by adding their email address below. This may be the same person you asked to complete questionnaires but it could also be someone different.

Click here to invite a friend

Accessibility

A- A A+

R A A

Launch ATbar (always?)

PWA

Abby Dunn

Raising Confident Children

Starter Module

Module: The Comfort Zone

Module: The Playful Parent

Module: Be Your Child's Emotion Coach

Module: Get More Good and Brave Behaviour

Module: Mind and

Raising Confident Children

Progress: 90%

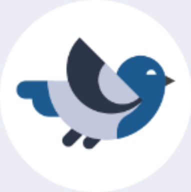

Starter Module

Welcome to the first module of this course. In this module, you will learn about how this course works and how to get the best out of it. You will then learn the basics about anxiety in children and adults.

Continue this module

Progress: 85%

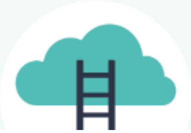

Module: The Comfort Zone

Progress Bar

Course progress: 82 %
