# Supplementary material for: Effectiveness of a Web-Based Intervention to Prevent Anxiety in the Children of Parents With Anxiety: Protocol for a Randomized Controlled Trial
Source: JMIR Res Protoc. 2022 Nov 10;11(11):e40707. doi: 10.2196/40707 (PMC9693706; doi:10.2196/40707)
Supplement: Multimedia Appendix 3 [file resprot_v11i11e40707_app3.docx]

## Measures

##

Child Measures (items)

SCAS: (38) (anxiety)/SCAS-Pre-school (28) (anxiety)

PSC: (17) (general mental health symptoms)

EQ-5D-Y (6) (health economic measure)

Parenting Behaviour Measures CPBQ: (104)

ADDITIONAL Mediator ITEMS: (31)

Parent Mental Health and Wellbeing Measures

SCARED-A: (71) (anxiety)

SWEMWBS: (7) (general wellbeing)

Co-respondent Measure

GAD-7: (7) (anxiety)

**SPENCE Child Anxiety Scale (SCAS)**

The Spence Children's Anxiety Scale was developed to assess the severity of anxiety symptoms in children aged 8-15.

39-items scored using a 4-point scale (0 = never, 1 = sometimes, 2 = often, 3 = always). Item 39 not used in this study.

1. My child worries about things
2. My child is scared of the dark
3. When my child has a problem, s(he) complains of having a funny feeling in his / her stomach
4. My child complains of feeling afraid
5. My child would feel afraid of being on his/her own at home
6. My child is scared when s(he) has to take a test.
7. My child is afraid when (s)he has to use public toilets or bathrooms
8. My child worries about being away from us / me
9. My child feels afraid that (s)he will make a fool of him/herself in front of people
10. My child worries that (s)he will do badly at school
11. My child worries that something awful will happen to someone in our family
12. My child complains of suddenly feeling as if (s)he can't breathe when there is no reason for this
13. My child has to keep checking that (s)he has done things right (like the switch is off, or the door is locked)
14. My child is scared if (s)he has to sleep on his/her own
15. My child has trouble going to school in the mornings because (s)he feels nervous or afraid
16. My child is scared of dogs
17. My child can't seem to get bad or silly thoughts out of his / her head
18. My child suddenly starts to tremble or shake when there is no reason for this.
19. When my child has a problem, s(he) complains of his/her heart beating really fast.
20. My child worries that something bad will happen to him/her
21. My child is scared of going to the doctor or dentist
22. When my child has a problem, (s)he feels shaky
23. My child is scared of heights (e.g. being at the top of a cliff)
24. My child has to think special thoughts (like numbers of words) to stop bad things from happening
25. My child feels scared if (s)he has to travel in the car, or on a bus or train
26. My child worries what other people think of him/her
27. My child is afraid of being in crowded places (like shopping centres, the movies, buses, busy playgrounds)
28. All of a sudden my child feels really scared for no reason at all
29. My child is scared of insects or spiders
30. My child complains of suddenly becoming dizzy or faint when there is no reason for this
31. My child feels afraid when (s)he has to talk in front of the class
32. My child’s complains of his / her heart suddenly starting to beat too quickly for no reason
33. My child worries that (s)he will suddenly get a scared feeling when there is nothing to be afraid of.
34. My child is afraid of being in small, closed places, like tunnels or small rooms
35. My child has to do some things over and over again (like washing his / her hands, cleaning or putting things in a certain order)
36. My child gets bothered by bad or silly thoughts or pictures in his/her head
37. My child has to do certain things in just the right way to stop bad things from happening
38. My child would feel scared if (s)he had to stay away from home overnight

2000 Susan H. Spence

**SPENCE Preschool Anxiety Scale (SCAS-P)**

The Preschool Anxiety Scale consists of 28 scored anxiety items (Items 1 to 28) that ask parents to report on the frequency of which an item is true for their child. Each item is rated on a 5-point scale (4 if the item is very often true, 3 if the item is quite often true, 2 if the item is sometimes true, 1 if the item is seldom true or if it is not true at all circle the 0). Item 29 (open-ended) is not included in the trial assessment battery.

1. Has difficulty stopping him/herself from worrying…
2. Worries that he/she will do something to look stupid in front of other people
3. Keeps checking that he/she has done things right (e.g., that he/she closed a door, turned off a tap)
4. Is tense, restless or irritable due to worrying…
5. Is scared to ask an adult for help (e.g., a preschool or school teacher)
6. Is reluctant to go to sleep without you or to sleep away from home…
7. Is scared of heights (high places)
8. Has trouble sleeping due to worrying
9. Washes his/her hands over and over many times each day
10. Is afraid of crowded or closed-in places
11. Is afraid of meeting or talking to unfamiliar people
12. Worries that something bad will happen to his/her parents
13. Is scared of thunder storms
14. Spends a large part of each day worrying about various things
15. Is afraid of talking in front of the class (preschool group) e.g., show and tell
16. Worries that something bad might happen to him/her (e.g., getting lost or kidnapped), so he/she won’t be able to see you again
17. Is nervous of going swimming.
18. Has to have things in exactly the right order or position to stop bad things from happening
19. Worries that he/she will do something embarrassing in front of other people
20. Is afraid of insects and/or spiders
21. Has bad or silly thoughts or images that keep coming back over and over
22. Becomes distressed about your leaving him/her at preschool/school or with a babysitter
23. Is afraid to go up to group of children and join their activities
24. Is frightened of dogs
25. Has nightmares about being apart from you
26. Is afraid of the dark
27. Has to keep thinking special thoughts (e.g., numbers or words) to stop bad things from happening
28. Asks for reassurance when it doesn’t seem necessary

1999 Susan H. Spence and Ronald Rapee

Pediatric Symptom Checklist (PSC-17)

17 items scored using a 3-point scale (0 = never, 1 = sometimes, 2 = often)

1. Feels sad, unhappy
2. Feels hopeless
3. Is down on self
4. Worries a lot
5. Seems to be having less fun
6. Fidgety, unable to sit still
7. Daydreams too much
8. Distracted easily
9. Has trouble concentrating
10. Acts as if driven by a motor
11. Fights with other children
12. Does not listen to rules
13. Does not understand other people’s feelings
14. Teases others
15. Blames others for his/her troubles
16. Refuses to share
17. Takes things that do not belong to him/her

Created by W Gardner and K Kelleher (1999), and based on PSC by M Jellinek et al. (1988) Formatted by R Hilt, inspired by Columbus Children’s Research Institute formatting of PSC-17

EQ-5D-Y Proxy

Five-item scale for which the child’s caregiver rates the health or wellbeing of the child on that day. They child’s overall health is also graded on a scale numbered from 0-100.

1. MOBILITY (walking about)

- No problems walking about
- Some problems walking about
- A lot of problems walking about

1. LOOKING AFTER HIM/HERSELF

- No problems washing or dressing him/herself
- Some problems washing or dressing him/herself
- A lot of problems washing or dressing him/herself

1. DOING USUAL ACTIVITIES (for example: going to school, hobbies, sports, playing, doing things with family or friends)

- No problems doing his/her usual activities
- Some problems doing his/her usual activities
- A lot of problems doing his/her usual activities

1. HAVING PAIN OR DISCOMFORT

- No pain or discomfort
- Some pain or discomfort
- A lot of pain or discomfort

1. FEELING WORRIED, SAD OR UNHAPPY

- Not worried, sad or unhappy
- A bit worried, sad or unhappy
- Very worried, sad or unhappy

1. Please indicate on the scale how you think the child’s health is TODAY.

THE CHILD'S HEALTH TODAY =

EuroQol Research Foundation

**Comprehensive Parenting Behavior Questionnaire 2-11 years**

104-items focused on parental interaction. Each is scored according to a 5-point scale (1 = Not applicable, 2 = Slightly appliable, 3 = Sometimes, but sometimes not applicable, 4 = Usually applicable, 5 = Completely applicable).

1. I play little tricks on my child
2. I constantly keep an eye on my child, to prevent him/her from getting hurt
3. I regularly play or talk with my child for at least 5 minutes, with full concentration on each other, just for fun
4. I give my child the feeling that he/she is a burden on me
5. When my child really misbehaves, I spank him/her
6. When my child doesn’t listen to me, I explain that I find that annoying
7. I play boisterously with my child
8. I do not look at my child when he/she has disappointed me
9. I show my child that I love him/her
10. I am often harsh towards my child
11. My child knows how to persuade me not to give out punishment when he/she has done something wrong
12. I reward my child or give him/her something extra if he/she behaves well
13. If my child finds something scary, I encourage him/her to carry on regardless
14. I never take my child to busy places
15. I give my child the feeling that I love him/her the way he/she is; I don’t want to change him/her
16. At times I totally have had it with my child, and reject him/her
17. When my child is too boisterous or noisy at home, I yell at him/her
18. When we go out, I prepare my child for this in advance
19. I encourage my child to approach unfamiliar people to ask them something
20. I cannot stand it when my child plays with things which make the house messy
21. I know exactly how to calm my child down when he/she is upset
22. I sometimes get very irritated when my child cries, and I do not hide it
23. I am often inconsistent in applying the rules I have made for my child
24. When my child has hit someone, I explain that he/she has hurt the other person
25. I encourage my child to be the best
26. I keep my child away from risky situations
27. I do fun activities with my child, such as handicraft or baking cookies
28. I am not easily pleased by my child
29. When I’m tense or irritable, I take it out on my child
30. I praise my child if he/she is being nice to other children
31. My child sometimes sees me horsing around with other people
32. I encourage my child to do things by him/herself
33. I show my love to my child by cuddling him/her, holding him/her and kissing him/her
34. When my child does something stupid, I react with irritation
35. When my child does not do what I ask, I often leave it at that
36. I make sure my child knows what is allowed and what is not
37. I regularly tease my child for fun
38. I don’t take my child out shopping because the fuss is too much for him/her
39. If my child does something naughty, I correct him/her, but at the same time I show that I still love him/her
40. When my child reacts differently from what I expected, I respond with disappointment
41. If my child does not do what I ask, even after repeated warnings, I slap him/her
42. When my child wants to touch something, he/she is not allowed to, I explain why it is not allowed
43. I almost never play rough and rowdy games with my child
44. If my child has hurt my feelings, I stop talking to him/her until he/she does me a favor
45. I comfort my child and show understanding when he/she is upset
46. My child hardly ever irritates me
47. I threaten to punish my child but then I fail to follow through
48. I reward or praise my child when he/she behaves properly, such as when he/she says ‘thank you’
49. If I see something that is new or exciting to my child, I encourage him/her to approach it
50. When my child climbs or clambers, I tell him/her that he/she should not climb too high because otherwise he/she might fall
51. Every day I play with child for a while, for example building something together or doing puzzles
52. When my child whimpers or moans, I shout at him/her
53. I set clear rules for my child
54. I encourage my child to perform for an audience by, for example singing a song, dancing, or doing something sporty
55. When I have a certain schedule in my head, my child has to cooperate
56. I often stroke my child’s head
57. When my child does something I don’t like, I often let it go
58. When my child has acted badly towards someone, I explain that it makes the other person sad
59. I challenge my child to contests, for instance running races or arm wrestling
60. I do not plan more than one outdoor activity per day because it might be too much for my child
61. If my son wants to dress up like a princess or my daughter wants to dress up like a pirate, I let him/her go ahead
62. When I’m stressed or tired, I react more severely to my child’s difficult behaviour
63. When my child helps, for example with clearing up toys, I give him/her a compliment
64. I show my child that I take risks
65. I encourage my child to do things in his/her own way
66. When my child asks something, I take my time when answering the question
67. The punishment I give my child depends on my mood
68. I give my child a warning when he\she appears to be starting to misbehave
69. As a prank, I sometimes give my child a real scare
70. During a risky activity I tell my child he/she should be careful
71. I often talk with my child
72. When my child goes too far, I slap him/her
73. I enjoy having pillow-fights with my child
74. When my child does something that’s not allowed, I pretend that he/she is not around
75. I often tell my child that I love him/her
76. When I ask my child to clean up and he/she does not do it, I eventually clean up myself
77. I encourage my child to do exciting things, such as jumping off high objects or climbing higher than he/she dares
78. I avoid doing things with my child that disturb his/her routine, for example when it means that bedtime will be later
79. I sometimes find it difficult to fully accept my child, including, his/her bad characteristics
80. If my child lingers when we are in a hurry, I shout at him/her
81. I encourage my child to say no if he/she doesn’t want something
82. I cannot stand it when my child suddenly wants something different from what we had planned
83. I immediately notice whether my child likes something or not
84. If my child makes a fuss when I say ‘no’, I give in to him/her
85. I encourage my child to compete against other children
86. I encourage my child to make his/her own decisions (RRR)
87. When my child is naughty and I am stressed, I shout at him/her
88. My child often sees me approach unfamiliar people
89. When my child does something that is not allowed, I usually refuse to talk to him/her until he/she behaves better
90. When saying ‘no’ doesn’t work, I offer my child a treat, so that he/she will behave.
91. I pretend that I’m going to eat my child’s sweets, for example his/her cookies or dessert
92. I want to monitor everything that my child is doing
93. When my child misbehaves, I grasp him/her roughly
94. I sometimes play ‘tag’ with my child: I chase after him/her and say in a low voice that I’m going to grab him/her
95. I ask my child for his/her opinion about things
96. When I have said that my child is not allowed to do something, I stick to it
97. I encourage my child to gain new experiences by, for example, eating something new or playing a new game
98. I often try to get my child to change
99. When my child misbehaves in public, I scold him/her
100. I encourage my child to stand up for himself/herself
101. When my child does not listen, I respond without getting angry (RRR)
102. I urge my child on when he/she is competing against other children
103. I show my child that I engage with situations that I find exciting or scary
104. If my child comes to me because he/she is having a minor quarrel, I make him/her sort it out by himself/herself

Comprehensive Parenting Behavior Questionnaire 4-6 years (CPBQ4-6) © 2011

Mirjana Majdandžić, Wieke de Vente, and Susan M. Bögels

**Additional mediator items: Your experience of being a parent**

Items devised by the study team focused on the intervention.

*22 items scored using a 6-point scale (strongly disagree, somewhat disagree, neither agree nor disagree, somewhat agree, strongly agree)*

1. It is important that my child believes the world is a fairly safe place.
2. It is important that my child believes he/she can cope with most things
3. It is important that my child believes bad things don’t usually happen to them
4. It is important that my child believes bad things don’t usually pop up out of the blue
5. It is important that my child believes he/she has some control over the things that happen to them
6. It is important that my child believes people are pretty nice really
7. It is important that my child believes other people respect them
8. I believe that the world is a fairly safe place
9. It is OK for my child to be scared sometimes
10. It is OK for my child to avoid things that frighten them
11. If my child became really scared of something I would know how to do a bravery ladder with them
12. I prefer that my child is supervised closely by an adult when they are engaging in risky play
13. I stop my child from doing rough play
14. It is OK for my child to be angry or sad sometimes
15. I notice my child’s feelings and talk to them about it
16. I can cope when my child is upset
17. I know how to use a star chart to help my child with a difficult behaviour
18. I try to be a perfect parent.
19. It is OK for my child to be a little stressed at times.
20. I let my child fail at things sometime
21. I am very protective of my child
22. If my child misses out on things because of my anxiety, I make sure they have those experiences in other ways

*Six items scored using a 5-point scale (always/ very often/ sometimes/ rarely/ never)*

1. I reward or praise my child when he/she is brave, such a when he/she faces up to
2. something they are scared of
3. When I praise or reward my child, I tell them clearly what it is for.
4. Sometimes I will take away a reward if my child is naughty after I’ve given it.
5. My child sees me feeling very frightened of things
6. I try to hide my anxiety from my child

*Four items scored using a 4-point scale (every day, most days, occasionally, never)*

1. My child gets enough sleep
2. My child gets enough exercise
3. My child has caffeine (coffee, tea, cola, energy drinks)
4. My child has a healthy diet

**SCARED-A**

71 items rated on a 3-point scale to indicate how frequently the symptoms have been experienced: “almost never”, “sometimes”, or “often”.

1. When I feel frightened, it is hard to breathe
2. I'm afraid of standing on a high peak (e.g. a tower) and look down.
3. I'm worried about my partner leaving me
4. I don't like to be with unknown people
5. When I see blood, I get dizzy
6. I want things to be in a fixed order
7. I get scared when I sleep away from home
8. I worry about others not liking me.
9. When I get frightened, I feel like passing out
10. I think that I will be contaminated with a serious disease
11. I am nervous.
12. I have strange thoughts that frighten me
13. I follow my partner wherever s/he goes.
14. People tell me that I look nervous
15. I feel nervous with people I don't know well.
16. I am afraid to visit the doctor.
17. I'm worried about the closeness of my relationship with my children
18. When I get frightened, I feel like I am going crazy
19. I'm worried about possible events than can separate me from my family
20. I am afraid to visit the dentist.
21. I worry about being as good as other people.
22. I am afraid of an animal that is not really dangerous
23. I get scared when there is thunder in the air.
24. I blush, sweat or tremble when I'm with others
25. I do things more than twice in order to check whether I did it right
26. I have frightening dreams about a very aversive event I once experienced.
27. I want things to be clean and tidy.
28. When I get frightened, it feels like things are not real.
29. I would feel scared if I had to fly in an airplane.
30. I have nightmares about something bad happening my family.
31. I worry about being away from my family.
32. I perform rituals that help me to get less scared of my thoughts.
33. I'm afraid I will make a fool of myself when I'm with others
34. When I feel frightened, my heart beats fast.
35. I am scared when I get an injection.
36. I am afraid of getting a serious disease.
37. I feel weak and shaky.
38. I've had nightmares about something bad happening to me.
39. I feel nervous when I go to a party.
40. I am so scared of a harmless animal that I do not dare to touch it.
41. I worry about things working out for me.
42. I doubt whether I really did something.
43. When I get frightened, I sweat a lot
44. I am a worrier.
45. I feel scared when I watch a medical operation on TV
46. I try not to think about a very aversive event I once
47. Suddenly I get really frightened for no reason at all.
48. I am afraid to be alone in my house
49. I get scared when I think back of a very aversive event I once experienced.
50. It is hard for me to talk to unfamiliar people.
51. When I am frightened, I feel like I am choking.
52. People tell me that I worry too much.
53. I don't like to be away from my family.
54. I am afraid of having anxiety (or panic) attacks.
55. I worry that something bad might happen to my family.
56. I am shy.
57. I have unwanted thoughts about hurting other people.
58. I worry about what is going to happen in the future.
59. When I get frightened, I feel like throwing up.
60. I worry about how well I do things.
61. I worry about sleeping alone.
62. I worry about things that happened in the past.
63. I'm afraid to ask a question in a group of people.
64. When I feel frightened, I get dizzy.
65. I get scared in small, closed places.
66. I have strange, scary thoughts that I prefer not to have.
67. I am afraid of the dark.
68. I have unbidden thoughts about a very aversive event I once experienced.
69. I am afraid of an animal that most people do not fear.
70. I don't like being in a hospital.
71. I feel nervous when I am with other children or adults and I have to do something while they watch me.

Van Steensel & Bögels, 2014.

**Short Warwick Edinburgh Mental Wellbeing Scale (SWEMWBS)**

Seven items each of which is rated on a 5-point scale: 5 if the item is true all of the time, 4 if the item is very often true, 3 if the item is true some of the time, 2 if the item is rarely true, 1 if the item is true none of the time.

1. I’ve been feeling optimistic about the future
2. I’ve been feeling useful
3. I’ve been feeling relaxed
4. I’ve been dealing with problems well
5. I’ve been thinking clearly
6. I’ve been feeling close to other people
7. I’ve been able to make up my own mind up about things

Warwick-Edinburgh Mental Well-being Scale (WEMWBS) © NHS Health Scotland, University of Warwick and University of Edinburgh, 2006, all rights reserved.

**The generalized anxiety disorder 7-item (GAD-7) scale.**

Seven-item scale to assess severity of anxiety symptoms using 4-point scale (0 = not at all, 1 = several days, 2 = more than half the days, 3 = nearly every day).

1. Feeling nervous, anxious or on edge
2. Not being able to stop or control worrying
3. Worrying too much about different things
4. Trouble relaxing
5. Being so restless that it is hard to sit still
6. Becoming easily annoyed or irritable
7. Feeling afraid as if something awful might happen

Spitzer RL, Kroenke K, Williams JB, et al. 2006
