# Supplementary material for: Effectiveness of a Web-Based Intervention to Prevent Anxiety in the Children of Parents With Anxiety: Protocol for a Randomized Controlled Trial
Source: JMIR Res Protoc. 2022 Nov 10;11(11):e40707. doi: 10.2196/40707 (PMC9693706; doi:10.2196/40707)
Supplement: Multimedia Appendix 4 [file resprot_v11i11e40707_app4.pdf]

The Kavli Trust

Bergen, Norway

Sharing all profits with good causes

KAVLI TRUST 2019 CALL

Reviewers' scores and comments

Application title: Preventing anxiety in the children of anxious parents"

Host institution: University of Sussex

Principal investigator: Sam Cartwright-Hatton, Professor of Clinical Child Psychology

REVIEWER #1

Score: 74% (26/35)

2nd Full Proposal Review Form

Please rank the following categories between poor and exceptional for this application:

1 = Poor, 2 = Weak, 3 = Fair, 4 = Good, 5 = Very good, 6 = Excellent, 7 = Exceptional

Relevance and expected impact	6
Scientific quality	4
Feasibility	5
Principal investigator/Research environment	6
Leadership	5

Comments:

This study proposes to conduct a large RCT of an online intervention with anxious parents to see if they can reduce anxiety in their children. The study is ambitious with many aims and sub-objectives, all of which are relevant for the selected evidence gap. A large sample is proposed, which will be

necessary to conduct the component analyses to optimize the intervention and increase efficiency. Some noted strengths of the proposal include:

(1) the preventive nature of the study,

(2) the inclusion of a second parent and the attention to the role that fathers play,

(3) using continuous (as opposed to diagnostic) measures of anxiety and

(4) using a measure of parent well-being.

Overall, a rigorous design is proposed, but there are two chief concerns: (1) the primary outcome (child anxiety symptoms) is solely based on parent report and parents are obviously not masked to condition, and (2) the six-month wait-list control design prevents the examination of longer- term follow up and the preventive effects are unlikely to be realized within 6 months. There were no exclusion criteria for children which increases the external validity of the intervention, but physical health problems and/or developmental disabilities might be important to assess and control for in analyses. No empirical support for the efficacy of the intervention and the estimated effect size were presented in the application. In addition, there was not enough information provided on the content of the prevention modules and how they are relevant for parents of both 2 and 11 year olds; the application was missing a developmental focus. There is a strong and well-rounded investigative team but the leadership plan lacked detail. Overall, the study has substantial promise to answer a number of key questions.

REVIEWER #2

Score: 77% (27/35)

2nd Full Proposal Review Form

Please rank the following categories between poor and exceptional for this application:

1 = Poor, 2 = Weak, 3 = Fair, 4 = Good, 5 = Very good, 6 = Excellent, 7 = Exceptional

Relevance and expected impact	5
Scientific quality	5
Feasibility	5
Principal investigator/Research environment	6
Leadership	6

Comments:

Relevance and Expected Impact

This research project will test the effectiveness of an online intervention to help anxious parents learn ways to support their children so they do not become anxious. Having an anxious parent is one of the stronger predictors of whether children become anxious themselves. Yet, few anxious parents seek mental health treatment that provides guidance around parenting behaviors. This research

project seeks to expand reach by providing such treatment online. It expects this approach to be especially important in involving fathers, who tend not to participate otherwise.

If successful, this research project could have very important ramifications. It could open a new avenue to preventing anxiety disorders in a high risk population of children. Because the intervention is online, it should be relatively easy to disseminate with fidelity and accessible to all families who might benefit from it.

Scientific Quality

This research project will rely on a very large sample of 1754 families. It will include a follow-up period of six months. This research project will take advantage of an innovative dismantling design to identify which components in the online intervention are effective in changing parents' and children's behaviors. This will allow the intervention to cull ineffective components and refine future iterations of the intervention.

Feasibility

This research project expects to have substantial rates of drop-out and non-completion of assessments. It will embed a secondary intervention to determine whether paying parents to complete measures at baseline will reduce that problem. It is not clear, though, why this research project hypothesizes that such a provision would be effective or that the relatively small payment would be sufficient to incentivize different behaviors so far into the future. It would be preferable if the research project simply paid participants more for completing the follow-up assessments. This research project might be better off focusing on its core aims and not being distracted by this peripheral issue. However, this research project will need to adopt a rigorous approach to understanding sample attrition, ensure that it has enough baseline information about families to understand who does and does not remain in the study and appropriately impute missing follow-up data.

REVIEWER #3

Score: 83% (29/35)

2nd Full Proposal Review Form

Please rank the following categories between poor and exceptional for this application:

1 = Poor, 2 = Weak, 3 = Fair, 4 = Good, 5 = Very good, 6 = Excellent, 7 = Exceptional

Relevance and expected impact	6
Scientific quality	4
Feasibility	6
Principal investigator/Research environment	7
Leadership	6

Comments:

The rationale and intervention are excellent; however, the use of a waitlist design is a significant weakness. Applicants highlight the lack of outcome assessor blinding, which is unavoidable, but creates significant risk of detection bias.

The component analysis is well thought through and a strength of the design; consideration of MOST design to further strengthen this component is recommended. More detail with regard to the proposed mediators (parent behaviour), rationale/justification for these and how these are measured would have been valuable. Similarly, justification of proposed moderators would have strengthened the proposal.

The potential of online interventions, and online trials have yet to be realised in terms of engagement and retention in interventions. Data collection with regard to who accesses information/webpage about the trial, and who of these actually engage in the trial, as well as back-end metrics of which modules are used and for how long will be important. The 'Study within Trial' will usefully add to the field about incentivising; however, this is probably not a scalable intervention in terms of understanding how to ensure engagement and retention in real-world roll out of these types of interventions.

Applicants suggest they are keeping questionnaires to a minimum to maximise retention; however, one of their measures is 71 items. How long does the proposed battery of measurement take to complete? Also interesting to note how Ethics committees will respond to the proposal to send follow-up SMS/email reminders – they are often concerned to make sure this is kept to a minimum.

There is mention in the analysis section of adverse outcomes but it is not clear what is being measured, nor how, in this regard until later in the application. One concern in psychotherapy trials is the potential impact of engaging in treatment that is called 'evidence based' but experiencing no benefits from it – how does that impact on a sense of hopelessness, and future treatment seeking. With regard to analysis there is no information about how missing data will be managed.

REVIEWER #4

Score: 89% (31/35)

2nd Full Proposal Review Form

Please rank the following categories between poor and exceptional for this application:

1 = Poor, 2 = Weak, 3 = Fair, 4 = Good, 5 = Very good, 6 = Excellent, 7 = Exceptional

Relevance and expected impact	6
Scientific quality	5
Feasibility	6
Principal investigator/Research environment	7
Leadership	7

Comments:

"This proposal addresses the evidence gap: Preventing anxiety in at-risk children. The project being proposed aims to test the effectiveness of an online parent-focused intervention to prevent anxiety in children of anxious parents and examine elements of the intervention. This online intervention is adapted from a face-to-face intervention which has been tested with an RCT and found to have positive outcomes. Additional aims include conducting an exploratory study of intergenerational transmission of anxiety, explore impact of co-parent anxiety on severity and parenting behaviors on child outcomes and test the effects payment for completion of measures on retention (a study within a study randomizing participants in intervention arm to payment/no payment of baseline measure).

The applicants propose a study that will be completed entirely online over three years. Participants are a parent (over 16) of a child age 2-11 with self report substantial levels of anxiety (current or lifetime). For each objective, the applicants provided information on primary and secondary (when applicable) outcomes and measures, sample size calculated using power analysis, and study design and analysis. Measures are validated measures, although there is some concern that they will only be collecting parent reported measures.

Each of the objectives seem to be well designed, although the applicants seem to be trying to do a lot in this application. The applicants seem to be proposing a complex design, with several studies within a study, but have described each quite well and made a compelling case for the purpose, rigor and feasibility, especially given the limited page range. The wait-list control design does not seem necessary and precludes longer term follow up.

Research environment and leadership appear to have substantive and project/grant management experience. The PI developed the intervention; however, with the intervention being online and all measures being self-reported and conducted online, concern regarding researcher/intervention developer bias is mitigated."

Oslo, June 14th

IDA SVEGE

Senior advisor

The Kavli Trust Programme on Health Research
